# Solitary coronary artery nourishing the entire heart

**DOI:** 10.4103/0256-4947.59378

**Published:** 2010

**Authors:** Bengi Yaymaci, Murat Ugurlucan, Murat Basaran, Ozer Selimoglu, Ali Kocailik, Senay Akyildiz, Orhan Coskun, Melih Us

**Affiliations:** From the Cardiovascular Surgery Clinic, Goztepe Safak Hospital, Istanbul, Turkey

## Abstract

Coronary artery anomalies are being more frequently diagnosed these days both because increasing numbers of patients are undergoing diagnostic studies and because advanced radiographic imaging methods are now commonly available. An isolated single coronary artery giving rise to the main coronary branches is a rare congenital anomaly. In this report we present a patient with a solitary coronary ostium, with both the left and right coronary artery systems arising from it, and then following their usual courses. This case was diagnosed incidentally during conventional angiography.

Coronary anatomy is an important issue during the management of a wide range of congenital and acquired cardiovascular pathologies. Knowledge of normal coronary physiology and of the variations in anatomy are vital prior to surgical interventions on the heart. Good operative outcome mostly depends on a thorough understanding of the coronary circulation.

Today, variations in coronary anatomy are frequently detected as a result of the availability of better screening systems and because an increasing number of patients are seeking solutions for cardiovascular disorders. Although variant conditions are infrequent^1^–^10^ there is much heterogenicity in the coronary vasculature, and as a result there is no well-established classification system in the literature and no clear-cut guidelines on whether to treat or not to treat these variant conditions. In this report, we present a 54-year-old patient with a solitary coronary ostium giving rise to the right and left main coronary arteries, with the latter supplying blood to the entire heart. Informed consent was obtained from the patient for publication of this case report.

## CASE

The patient was a 54-year-old male who presented to the clinic with chest pain and dyspnea on exertion. He had history cigarette smoking (1 pack/day for 30 years). His past history was otherwise unremarkable. Family history included death of the father at the age of 45 years, most probably due to a myocardial infarction. However, his elder male siblings were alive and free of any symptoms. His electrocardiography (ECG) was normal. He underwent a stress ECG, which had to be stopped at the third minute of maximal effort capacity because the patient reported fatigue and dyspnea. Angiography was decided upon for further diagnostic workup.

**Figure 1:**
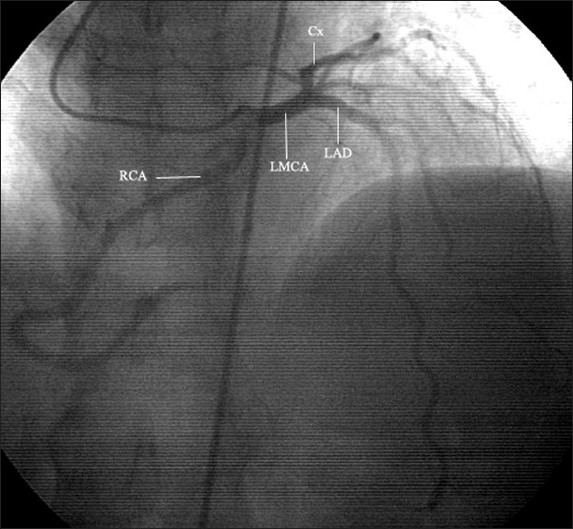
Coronary angiography, right anterior oblique view with a caudal projection. Note the single coronary ostium and the right coronary artery and left main coronary artery originating from the solitary vessel. LMCA: Left main coronary artery; LAD: left anterior descending coronary artery; RCA: right coronary artery; Cx: circumflex coronary artery.

**Figure 2:**
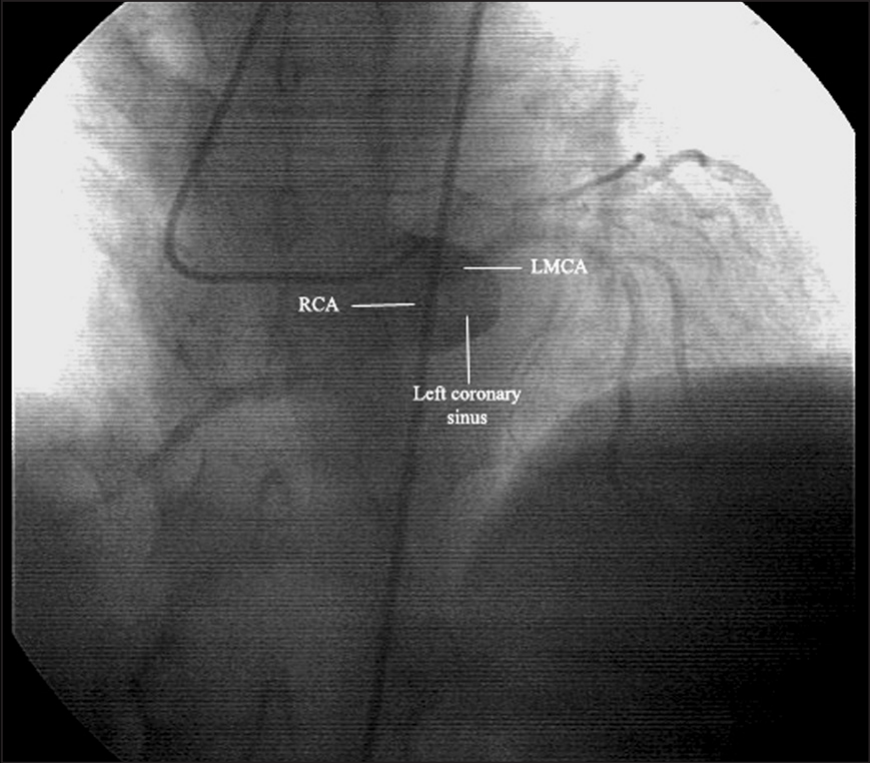
Angiographic imaging of left coronary sinus showing a single coronary ostium. LMCA: Left main coronary artery; RCA: right coronary artery.

Coronary angiography was performed using the traditional Judkings method.^11^ The left coronary system was completely normal. Moreover, it gave rise to the right coronary artery (Figures 1 and 2). Another check to visualize the right coronary artery in the right coronary sinus did not reveal the right coronary ostium as expected. Ventriculography indicated minor inferior wall dyskinesia. Echocardiography confirmed the inferior wall motion abnormality and revealed mild aortic and mitral insufficiencies.

We recommended aspirin 300 mg daily and cessation of smoking and advised him to come for yearly follow-ups. Further nuclear or radiographic studies such as coronary CT angiography or MR imaging were not done.

## DISCUSSION

Coronary artery anomalies are rare. They are seen in 0.3% to 1.3% of patients undergoing coronary angiography^1^ and in 0.17% of routine autopsy studies.^2^ In the younger age groups, they may be responsible for 4% to 15% of cases of sudden death.^2^,^3^

An isolated single coronary artery is a solitary coronary vessel arising from the ascending aorta, giving rise to the major coronary branches and thus nourishing the entire heart. In effect, both the right coronary artery and the left main coronary artery arise from a single aortic sinus.^6^ The anomaly is usually diagnosed incidentally during coronary artery angiograms or on postmortem evaluations. The incidence of single coronary artery is approximately 0.019% to 0.4% in the population.^4^–^6^ They reportedly constitute <3% of all coronary anomalies.^1^ They are frequently found in association with other congenital cardiac lesions. In adult subjects, the clinical interest relates to their occasional association with myocardial ischemia, congestive heart failure, and/or sudden death. Additionally, the presence of coronary artery anomalies may pose special problems during coronary angiography, interventional procedures, and coronary artery bypass surgery.

There is no gender preference for coronary artery anomalies.^3^,^4^ The well-known ALCAPA (anomalous origin of left main coronary artery from the pulmonary trunk) syndrome, when significant, may result in symptoms during young adulthood; otherwise the syndrome is generally clinically silent and is discovered only incidentally during diagnostic studies for other pathologies. The exact incidence of clinical events due to coronary artery anomalies is not known as most cases are clinically silent and the subject's quality of life and life expectancy are unaffected.

A single coronary artery may be diagnosed by various imaging modalities, including CT, MR imaging, echocardiography, and conventional coronary angiography.^6^,^7^ The advances in radiologic diagnostic methods, especially in CT and MR imaging, has allowed the origin and course of anomalous coronary arteries to be demonstrated with excellent resolution. However, coronary angiography is still the gold standard diagnostic tool, enabling simultaneous percutaneous interventions for treatment of associated or non-associated coronary lesions.^7^

A single coronary artery may appear in various forms. Smith, in the mid-20^th^ century, classified single coronary artery anomalies into three major groups.^9^ In the first type, the single coronary artery follows the course of the right coronary artery, continues into the circumflex artery and then as the left anterior descending coronary artery; alternatively, there may be a single left main artery that branches into the left anterior descending artery and circumflex artery, the latter extending across the crux to form the right coronary artery. In the second type, after its origin the main trunk divides into the right and left main arteries (as in our patient) or into right coronary artery, left anterior descending coronary artery, and circumflex artery. In the last type, the single coronary artery branches atypically and so there is little similarity between the coursing of the three major arteries.

There is no consensus regarding the risk for atherosclerosis in casees of single coronary artery. The literature includes variable reports. Porto et al. claim that there is an increased risk of atherosclerosis in the case of a single coronary artery, which may result from acute-angle take-off malformation.^10^ Coronary spasm,^12^ angina pectoris, and myocardial infarction are also reported in the absence of coronary stenosis.^13^ The literature includes reports of percutaneous angioplasty and stenting in single coronary artery.^10^ In contrast, according to Garg et al.^14^ single coronary artery anomaly is not associated with increased risk for the development of atherosclerotic coronary artery disease. In our case, the patient presented with chest pain and dyspnea on exertion. Additionally, there was minor inferior wall dyskinesia, which may be also associated with coronary anomaly.

In conclusion, a single coronary artery is a rare congenital anomaly. In the case presented, the right coronary sinus ended blindly. All the coronary arteries originated from a solitary coronary vessel and then followed their usual courses in the myocardium. We believe with the presentation.
